# Association of Vaping and Respiratory Health among Youth in the Population Assessment of Tobacco and Health (PATH) Study Wave 3

**DOI:** 10.3390/ijerph18158208

**Published:** 2021-08-03

**Authors:** Christie Cherian, Eugenia Buta, Patricia Simon, Ralitza Gueorguieva, Suchitra Krishnan-Sarin

**Affiliations:** 1 Pediatric Pulmonology, Yale School of Medicine, Yale University, New Haven, CT 06510, USA; christie.cherian@gmail.com; 2 Yale Center for Analytical Sciences, Yale School of Public Health, Yale University, New Haven, CT 06510, USA; eugenia.buta@yale.edu; 3 Department of Psychiatry, Yale School of Medicine, Yale University, New Haven, CT 06510, USA; p.simon@yale.edu; 4 Department of Biostatistics, Yale School of Public Health, Yale University, New Haven, CT 06510, USA; ralitza.gueorguieva@yale.edu

**Keywords:** electronic nicotine products (ENP), adolescents, pulmonary, wheezing, cough, public policy

## Abstract

The purpose of this study is to evaluate the association of electronic nicotine product (ENP) use and its respiratory manifestations in a nationally representative sample of adolescents in the US. Cross-sectional evidence from 9750 adolescents in wave 3 (October 2015–October 2016) of the Population Assessment of Tobacco and Health (PATH) survey was used. Adjusting for demographics, lifetime number of cigarettes and cigars used, home rules about tobacco use, and tobacco used by other household members, we used logistic regression models to examine associations between ENP use and its respiratory manifestations in the past year. Among 9750 adolescents, 12% (*n* = 1105) used ENP in the past year. Compared to non-users, past-year ENP-users had 37% higher odds of wheezing in general (Adjusted Odds Ratio (AOR) = 1.37, 95% Confidence interval (CI): 1.11–1.71, *p* = 0.005) and higher odds of wheezing 4–12 times or >12 times per year versus no wheezing (AOR = 1.57, 95% CI: 1.01–2.46, *p* = 0.05 and AOR = 2.58, 95% CI: 1.04–6.41, *p* = 0.04, respectively). Additionally, odds of dry cough at night were 23% higher among ENP-users than among non-users (AOR = 1.23, 95% CI: 1.04–1.46, *p* = 0.02). There was no association between past-year ENP use and exercise-induced wheezing or asthma diagnosis. Among those with asthma, there was no evidence of an association between ENP use and long-acting inhaler or quick-relief inhaler use. ENP use among adolescents is associated with increased frequency of wheezing and dry cough. Early recognition of pulmonary clinical manifestations among young ENP users should be critical considerations in regulatory and prevention efforts to protect public health, and clinical efforts to prevent progression to serious pulmonary complications.

## 1. Introduction

Electronic nicotine product (ENP) use, also known as vaping, has seen an increase in recent years especially among adolescents [1] and has been declared an epidemic among youth in the United States [2]. While evidence on the safety and toxicity of e-cigarettes is emerging, many adolescents believe that e-cigarettes are not harmful or minimally harmful [3]. However, there is extensive preclinical evidence that suggests that nicotine, a main component of many e-cigarettes, can have detrimental effects on the developing brain [4]. Adolescent vaping is also a risk factor for future cigarette smoking and may serve as a conduit to cigarette smoking and other addiction patterns more so in youth than in adults [5]. Thus, it is critical to develop a better understanding of the health effects of e-cigarette exposure among youth.

From a respiratory perspective, evidence suggests that exposure to e-cigarettes even for 5 min can increase airway resistance [6]. E-cigarettes also increase oxidative stress, interfere with lung development, increase the production of inflammatory chemicals, and impair alveolar macrophage activity, which disables key protective cells in the lung that keep the air spaces clear of potentially harmful particles [7,8]. Clapp and colleagues have suggested that common flavoring agents that are present in e-liquids are chemically similar to known airway irritants and sensitizers, and have been reported to cause occupational asthma [9]. Multiple cases of e-cigarette, or vaping, product use-associated lung injury (EVALI) were observed in 2019 in the US [10] with approximately 15% of these patients being under 18 years of age [11]. EVALI cases have been associated with pneumonitis, acute eosinophilic pneumonia [12], organizing pneumonia, lipoid pneumonia [13,14,15], diffuse alveolar hemorrhage [16], acute respiratory distress syndrome (ARDS) [17], hypersensitivity pneumonitis [17], and giant-cell interstitial pneumonitis [18,19], reflecting a diverse spectrum of disease processes. The pathophysiology of EVALI and other respiratory conditions seen with e-cigarette use is poorly understood [20]; however, it is believed that Vitamin E acetate found in some THC-containing e-cigarette, or vaping, products, is strongly linked to EVALI [11]. Early identification of subacute respiratory symptoms that could progress to lung dysfunction could advance the understanding and treatment of ENP-associated lung injuries.

There is growing literature on the influence of vaping on respiratory outcomes. Adult dual cigarette smokers and e-cigarette users were observed to have higher odds of multiple respiratory symptoms when compared to those who do not use e-cigarettes [21,22]. Among adults, an analysis of Wave 2 (2014–2015) evidence from the US Population Assessment of Tobacco and Health (PATH) study demonstrated that vaping is associated with an increased risk of wheezing [23,24]. Similarly, among youth, a population study of 35,904 high school students in Korea in 2014 demonstrated that e-cigarette users had an increased association with severe asthma symptoms [25]. In 2012–2013, a study in Hong Kong among 45,128 adolescents found that e-cigarette use is associated with increased odds of chronic cough and phlegm [26]. Surveys among 6089 adolescents in Hawaii (2015) [27] and 36,085 adolescents in Florida (2012) [28] demonstrate that e-cigarette use is associated with a diagnosis of asthma. In 2014, the Southern California Children’s Health Study among 502 e-cigarette users in 11th and 12th grade showed that the odds of chronic cough, phlegm, or bronchitis were increased by almost two-fold among both past users and current users; however, there were no statistically significant associations of e-cigarette use with wheezing [29]. In 2020, a study in Kuwait among 369 high school e-cigarette users demonstrated that e-cigarette use and their household exposure to second-hand aerosol from e-cigarettes were associated with asthma symptoms [30]. Most recently, a scoping review, which reviewed the literature to date and reported on the adverse pulmonary effects of ENP use among youth and adults, concluded that the evidence of pulmonary effects in adolescent populations was rather limited to evidence obtained in certain US states or to countries outside of the US, highlighting the need for more evidence from larger, nationally representative studies among youth [31]. Therefore, we examined the association between ENP use and multiple respiratory symptoms using a nationally representative dataset from adolescents in the US. Further, we examined these relationships during 2015–2016, following a significant rise in youth ENP use rates from 2013–2015 [32,33].

Using a nationally representative sample of youth from the US PATH study Wave 3 data, we investigated e-cigarette use or electronic nicotine product (ENP) use/vaping in the last 12 months and its association to respiratory symptoms during the same period (i.e., in the last 12 months). We examined respiratory symptoms such as wheezing, exercise-induced wheezing, and dry cough, as well as an asthma diagnosis, or use of long-acting and/or short-acting inhalers among those who already have asthma. We hypothesized that e-cigarette use would be associated with increased respiratory symptoms among these adolescents, and, in those diagnosed with asthma, with increased use of a long-acting and short-acting inhaler.

## 2. Methods

### 2.1. Study Population

We used the Population Assessment of Tobacco and Health (PATH) study database, which is one of the largest nationally representative longitudinal studies of tobacco use and health in the United States. The PATH Study was launched in 2011 to inform Food and Drug Administration’s regulatory activities under the Family Smoking Prevention and Tobacco Control Act. Details regarding the conduct of the PATH study can be found via the online database [34].

We used the public version of Wave 3 PATH dataset; this evidence was collected from October 2015 to October 2016. We were interested in studying whether vaping was related to poorer respiratory outcomes among youth. Since the primary respiratory clinical outcomes (described below) were assessed over the past 12-month period, we considered e-cigarette use during a similar time-period, i.e., the past 12 months. There were 11,814 youth interviewed at Wave 3, out of which *n* = 9769 “continuing youth” (i.e., youth that were also interviewed in Wave 1 or 2) were considered for these analyses, as the other *n* = 2045 “new baseline youth” (i.e., youth who had their first PATH interview at Wave 3) were not asked the questions about past 12-month use of electronic nicotine products. We excluded *n* = 19 participants with missing data on the past 12-month use of electronic nicotine product; thus, the final sample included 9750 youth.

### 2.2. Study Variables

#### 2.2.1. Respiratory/Clinical Outcomes

Survey participants were asked whether they experienced the following in the past 12 months: wheezing or whistling in chest (yes/no); number of times had wheezing in chest (none, 1 to 3, 4 to 12, more than 12); chest sounded wheezy during or after exercise (yes/no); dry cough at night (yes/no); been told by a doctor, nurse or other health professional that you have asthma (yes/no). Those who were ever diagnosed with asthma were additionally asked whether, in the past 12 months, they used a quick-relief inhaler (yes/no) and whether they used a controller/long-acting inhaler (yes/no).

#### 2.2.2. Use of Electronic Nicotine Products

Participants were asked if, in the past 12 months, they used an electronic nicotine product, even one or two times (yes/no).

#### 2.2.3. Covariates

We covaried for a number of variables that have been shown in previous studies to influence respiratory outcomes from cigarettes to e-cigarettes [23,29].

Frequency of combustible tobacco product use in lifetime. Participants were also asked how many of each of the following products—cigarettes, traditional cigars, filtered cigars, and cigarillos—they smoked in their entire life (“1 or more puffs but never a whole one”, “1”, “2 to 10”, “11 to 20”, “21 to 50”, “51 to 99”, “100 or more”). Those who never smoked a product were included in a “none” category. We combined the traditional cigars, filtered cigars, and cigarillos variables into a single cigar variable by taking the maximum. We collapsed some categories due to low counts so that the final variables had the following four categories: “none”, “at most 1”, “2 to 99”, “100 or more”.

Tobacco used by other household members. Participants were asked if anyone who lives with them now does any of the following: smoke cigarettes; use smokeless tobacco; smoke cigars, cigarillos, or filtered cigars; or use any other form of tobacco. The variable had 4 categories: (1) Cigarettes, cigars, cigarillos, or filtered cigars; (2) E-products exclusively; (3) Other tobacco products, including smokeless, snus, and hookah; (4) No one living in the home uses tobacco.

Rules about using combustible tobacco inside home. Participants were asked: “Think about everyone who might be in your home including children, adults, visitors, guests, or workers. For tobacco products that are burned, such as cigarettes, cigars, pipes, or hookah, which statement best describes the rules about smoking a tobacco product inside your home?” The variable included 3 categories: (1) It is not allowed anywhere or at any time inside my home; (2) It is allowed in some places or at some times inside my home; (3) It is allowed anywhere and at any time inside my home.

Socio-demographics. Youth participants reported data on their sex (male, female), age (12–14, 15–17 years), Hispanic origin (yes/no), and race (White, Black, or other). The Hispanic origin and race variables were combined into a race/ethnicity variable with four levels: non-Hispanic White, Hispanic, non-Hispanic Black, or non-Hispanic Other. We also included parental education as a covariate; this information was obtained from the parental interview. Parents were asked what is the highest grade or year of school that they have completed (options included: less than high school, GED, high school graduate, some college (no degree) or associates degree, bachelor’s degree, advanced degree).

### 2.3. Statistical Analysis

We used univariable and multivariable logistic regression models to estimate the unadjusted and adjusted association between the primary predictor (electronic nicotine product (ENP) use, i.e., vaping in the past 12 months, yes/no) and whether the participant experienced each one of the following respiratory outcomes in the past 12 months (yes/no): wheezing/whistling; chest sounded wheezy during or after exercise; dry cough at night; participant was told by a doctor, nurse, or other health professional that he/she has asthma. For the number times wheezing in the past 12 months (none, 1 to 3, 4 to 12, more than 12), we used a multinomial logit model. The multivariable models were adjusted for the following covariates: age (12–14 vs. 15–17), sex (female vs. male), race/ethnicity (non-Hispanic White, non-Hispanic Black, non-Hispanic Other, Hispanic), parent education (less than high school, GED, high school graduate, some college (no degree) or associates degree, bachelor’s degree, advanced degree), tobacco used by other household members (4 categories), rules about using combustible tobacco inside the home (3 categories), number of cigarettes used in entire life (none, at most 1, 2 to 99, 100 or more), and number of cigars used in entire life (none, at most 1, 2 to 99, 100 or more). Similarly, we investigated the association between vaping and asthma medication use (quick-relief inhaler and controller/long-acting inhaler) in the past 12 months by using logistic regression models, but these analyses were restricted to the subsample of participants who were ever diagnosed with asthma. Results from all models are presented as odds ratios (OR), adjusted odds ratio (AOR), and their 95% confidence intervals (CI). We performed complete-case analyses as the amount of missing data was relatively low (models excluded at most 13% participants with missing data). All results are weighted (except frequencies (N’s)) using the single wave weights at Wave 3 and take into consideration the complex design of the PATH survey [34].

## 3. Results

### 3.1. ENP Use and Demographics

There was a total of 9750 (out of 9769) youth participants (ages 12–17) who had non-missing data on past 12-month ENP use. Overall, about half of these participants were male (51.4%), about half (53.9%) were non-Hispanic white, and 59.7% were in the older age group (15 to 17 years old) (Table 1). In this sample, 12% (*n* = 1105) of the participants used an ENP in the past 12 months. In comparison to participants who did not use ENP in the past 12 months, those who used ENP in the past 12 months were older, were more likely to be non-Hispanic whites, and used more cigarettes and cigars over their lifetime (Table 1).

### 3.2. E-Cigarette Use and Respiratory Outcomes

**Figure 1:**
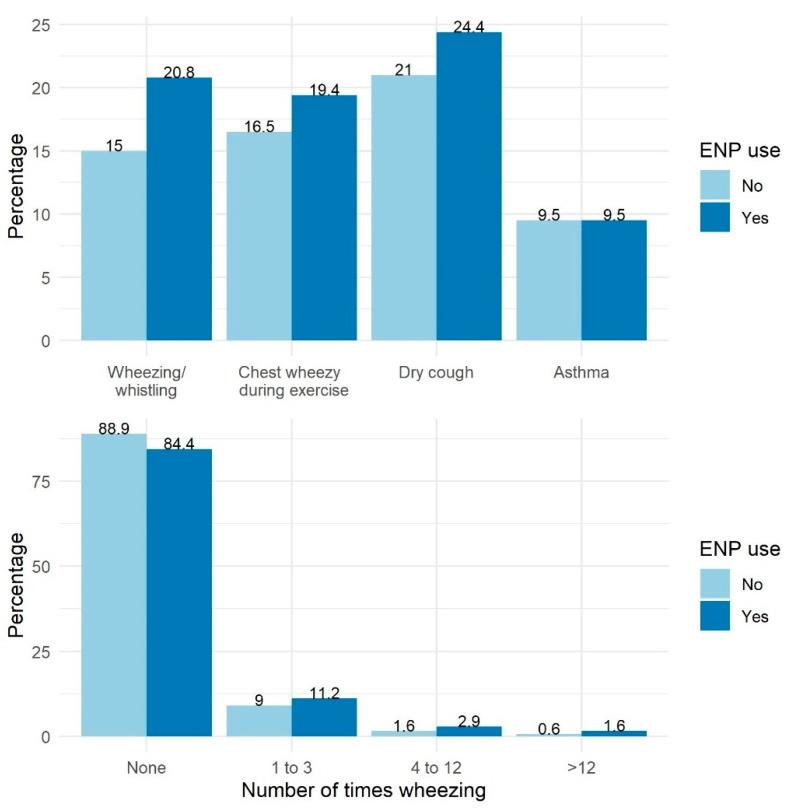
Respiratory outcomes (%) with and without electronic nicotine product (ENP) use in the past 12 months.

The percentage of subjects who experienced respiratory outcomes with and without ENP use are noted in Figure 1. In unadjusted (univariable) models (Table 2), using ENP in the past 12 months was associated with a higher likelihood of experiencing all considered respiratory outcomes (wheezing/whistling, number times wheezing, dry cough at night, and chest sounding wheezy during or after exercise) in the past 12 months (*p* < 0.05), except being told by a health professional that they had asthma (*p* = 0.95).

After adjusting for covariates (Table 2), wheezing/whistling, number times wheezing, and dry cough at night were still associated with ENP use (*p* < 0.05). However, there was no longer evidence of an association between “chest sounding wheezy during or after exercise” and ENP use (*p* = 0.18). The adjusted odds of reporting wheezing/whistling were 37% (95% CI 11% to 71%) higher in those who used ENP than in those who did not use ENP (*p* = 0.005). The adjusted odds of reporting wheezing 4 to 12 times or >12 times rather than no wheezing were higher in those who used ENP than in those who did not use ENP (AOR = 1.57, 95% CI 1.01–2.46, *p* = 0.05 and AOR = 2.58, 95% CI 1.04–6.41, *p* = 0.04, respectively). The adjusted odds of dry cough at night were 23% higher in those who used ENP than in those who did not use ENP (AOR = 1.23, 95% CI 1.04–1.46, *p* = 0.02). For the asthma outcome (i.e., participant being told by a health professional that they had asthma), the adjusted model (as in the unadjusted one) showed no evidence of an association with ENP use.

### 3.3. Association between Electronic Nicotine Product Use and Asthma Medication Use among Participants Who Ever Had Asthma

There were 1917 participants who ever had asthma. Twelve percent (*n* = 228) of these participants used ENP in the past 12 months. The percentage of participants who used a controller/long-acting inhaler was 12.5% in those who used ENP and 12.7% in those who did not use ENP. The percentage of participants who used a quick-relief inhaler was 22.4% in those who used ENP and 23.5% in those who did not use ENP.

The estimated unadjusted and adjusted associations between ENP use and asthma medication use among participants who ever had asthma are presented in Table 3. There was no evidence of an unadjusted (OR = 0.98, *p* = 0.93) or adjusted (AOR = 1.04, *p* = 0.88) association between vaping and controller/long-acting inhaler use. Similarly, there was no evidence of an unadjusted (OR = 0.94, *p* = 0.67) or adjusted association (AOR = 1.08, *p* = 0.62) between vaping and quick-relief inhaler use.

## 4. Discussion

This study aimed to understand the association between vaping and its pulmonary manifestations among youth during 2015–2016 following a significant rise in national rates of youth ENP use in the US. Using evidence from PATH, a nationally representative US dataset, we observed that youth who reported ENP use in the past 12 months, when compared with youth who did not use ENP in the past 12 months, had higher odds of wheezing in general, as well as of wheezing 4–12 times or >12 times per year rather than no wheezing. We also found that the odds of dry cough at night were higher among past 12-month vapers than among non-vapers. Furthermore, we performed a sensitivity analysis with models also adjusted for current cigarette use and current cigar use (in addition to all the other predictors); conclusions regarding the association between ENP use and respiratory outcomes stayed the same. We also found no evidence that the association between ENP use and respiratory outcomes is modified by tobacco used at home by other household members. Our evidence partially supports our hypothesis that ENP use among youth is associated with pulmonary manifestations as evidenced by cough and wheezing. There was no evidence of an association between e-cigarette use and exercise-induced wheezing or asthma diagnosis. Among those with asthma, there was no evidence of an association between e-cigarette use and long-acting inhaler use or quick-relief inhaler use. This may be secondary to a smaller sample size of youth with asthma. It is also possible that some of these respiratory outcomes may take a longer time to manifest with continued and higher frequency of ENP use and therefore warrant further investigations using evidence obtained after 2016.

Our findings suggest that perhaps airway inflammation as evidenced by wheezing, and bronchospasm as evidenced by cough, may be the initial symptoms related to e-cigarette exposure; however, this cannot be determined by the current study design. Although the exact mechanism of vaping-induced lung injury is not clear [11,35,36], one proposed molecular mechanism suggests lung injury may occur due to inhaled aerosols from e-cigarettes which accumulate in lung alveoli that may change the phenotype and function of alveolar macrophages which are responsible for clearing the airway of debris. This could lead to poor airway clearance and subsequent airway inflammation with the assistance of polymorphonuclear cells and airway epithelial cells that become irritated by the vapor leading to lung injury [36].

Our results are clinically relevant as they demonstrate an association between ENP use and worse respiratory outcomes, which may have implications for longer term effects. When clinicians evaluate respiratory complaints such as wheezing and cough among youth, they should also assess vaping history. Moreover, once vaping history is confirmed, specific questions regarding type of ENP, e-liquid type, flavorings, presence of THC/nicotine, and vaping frequency should also be assessed and addressed. This information can help us in understanding consistencies among patient symptom presentations. Subsequent education regarding the youth-specific dangers of vaping and counseling to assist youth with vaping cessation should also be provided.

While this study helps us understand the pulmonary manifestations of vaping in a large nationally representative sample of youth, some limitations should be noted. There may be recall bias as we used self-report assessments, and we are limited by the manner in which PATH questions are asked. We used past 12-month tobacco product use to match the 12-month assessment time frame of the respiratory outcomes, and we could not take into account variation of use within this time frame, or examine long-term effects. Since this study is observational and uses cross-sectional data, we cannot establish causality. 

To our knowledge, there are limited longitudinal studies in adults and none in adolescents. One adult study found increased risk of respiratory disease (COPD, bronchitis, emphysema, and asthma) among e-cigarette users that was even worse among dual users during just a 3-year time frame [37]. To understand the long-term effects of e-cigarette exposure on respiratory symptoms and the development of asthma, we will need multiple years of detailed and consistently defined data on vaping and on respiratory outcomes. Additional studies which focus on the mechanism of pulmonary toxicity as well as the clinical manifestations of vaping-induced lung injury will be helpful to understand the continuum of this disease process. Furthermore, since epigenetics may play a role in respiratory outcomes [38,39], future studies which take individual phenotypes into consideration, and its effect on respiratory outcomes will be interesting to examine. Finally, since youth use e-cigarettes to administer nicotine and marijuana [40], future studies should examine the influence of vaping marijuana versus vaping nicotine, taking into account the intensity of vaping and type of device used and how this influences respiratory outcomes.

## 5. Conclusions

Our evidence suggests that e-cigarette use among youth is associated with an increased likelihood of cough and wheezing. Previously published studies on pulmonary effects of vaping in adolescent populations are limited to evidence obtained in certain US states or to countries outside of the US. However, our study is unique as it has one of the largest sample sizes and is the first nationally representative study among youth in the US that examines ENP use and its association to multiple variables including cough, wheezing, exercise-induced wheezing, diagnosis of asthma, and the use of long-acting and short-acting inhalers, while controlling for a number of variables that have been shown in previous studies to influence respiratory outcomes. Understanding these associations may have implications on longer term respiratory effects and may help guide future studies. Our results can be used to educate and empower clinicians, parents, and adolescents about potential concerns related to e-cigarette use behaviors and highlight the crucial importance of prevention and cessation interventions addressing these behaviors. Our results also suggest that the FDA should consider including examinations of early subacute clinical symptoms of pulmonary toxicity among youth when considering the impact of e-cigarettes and vaping products on public health.

## Figures and Tables

**Table 1:** Descriptive statistics of PATH Wave 3 continuing youth overall and by electronic nicotine product use in past 12 months.

Variables	Overall (*n* = 9750)		Used ENP in Past 12 Months (*n* = 1105)		Did Not Use ENP in Past 12 Months (*n* = 8645)		*p*-Value Comparing the 2 Groups
	*n*	%	*n*	%	*n*	%	
**Age**							<0.0001
12 to 14 years old	3948	40.3%	237	20.4%	3711	42.9%	
15 to 17 years old	5802	59.7%	868	79.6%	4934	57.1%	
**Sex**							0.34
Male	5008	51.4%	581	52.6%	4427	51.2%	
Female	4717	48.6%	520	47.4%	4197	48.8%	
**Race/ethnicity**							<0.0001
Non-Hispanic White	4457	53.9%	578	61.5%	3879	52.8%	
Non-Hispanic Black	1267	13.3%	77	6.9%	1190	14.1%	
Non-Hispanic Other	899	9.6%	123	9.8%	776	9.6%	
Hispanic	2841	23.2%	304	21.8%	2537	23.4%	
**Parent education**							<0.0001
Less than High School	1432	12.2%	137	10.1%	1295	12.5%	
GED	421	3.9%	51	4.2%	370	3.9%	
High school graduate	1749	17.4%	215	18.9%	1534	17.2%	
Some college (no degree) or associates degree	3055	31.8%	408	38.2%	2647	30.9%	
Bachelor’s degree	1793	21.1%	176	18.7%	1617	21.4%	
Advanced degree	1092	13.7%	96	9.9%	996	14.2%	
**Tobacco used by other household members**							<0.0001
Cigarettes, cigars, cigarillos, or filtered cigars	2572	25.9%	409	36.0%	2163	24.6%	
E-products exclusively	169	1.8%	42	3.8%	127	1.6%	
Other tobacco products, including smokeless, snus, and hookah	373	4.1%	60	5.7%	313	3.8%	
No one living in the home uses tobacco	6509	68.2%	583	54.5%	5926	70.0%	
**Rules about using combustible tobacco inside home**							<0.0001
It is not allowed anywhere or at any time inside my home	7965	82.9%	821	76.5%	7144	83.7%	
It is allowed in some places or at some times inside my home	1004	10.3%	159	13.5%	845	9.9%	
It is allowed anywhere and at any time inside my home	664	6.8%	114	10.0%	550	6.4%	
**Lifetime number cigarettes used**							<0.0001
None	8371	88.3%	560	50.3%	7811	93.4%	
At most 1	467	5.0%	164	15.7%	303	3.5%	
2 to 99	405	4.5%	235	22.5%	170	2.0%	
100 or more	207	2.3%	123	11.5%	84	1.0%	
**Lifetime number cigars used**							<0.0001
None	8890	95.0%	823	77.8%	8067	97.3%	
At most 1	209	2.3%	81	7.5%	128	1.6%	
2 to 99	201	2.3%	129	12.9%	72	0.9%	
100 or more	38	0.4%	21	1.8%	17	0.2%	

Note: ENP electronic nicotine product. N’s are unweighted, and % are weighted. *p*-values are from weighted chi-squared tests. Overall *n* = 9750 does not include 19 participants missing information on past 12-month electronic nicotine product use.

**Table 2:** Unadjusted and adjusted association between electronic nicotine product use in past 12 month and respiratory outcomes (*n* = 9769).

	Unadjusted Results			Adjusted Results		
Outcome	OR (95% CI)	*p*-Value	*n* in the Model	AOR (95% CI)	*p*-Value	*n* in the Model
**Wheezing/whistling in chest past 12 months**	1.49 (1.28, 1.74)	<0.0001	9628	1.37 (1.11, 1.71)	0.005	8536
**Number times wheezing in chest past 12 months vs. none**			9638			8531
1 to 3	1.31 (1.06, 1.61)	0.01		1.23 (0.93, 1.63)	0.14	
4 to 12	1.93 (1.31, 2.84)	0.001		1.57 (1.01, 2.46)	0.05	
>12	2.94 (1.49, 5.79)	0.002		2.58 (1.04, 6.41)	0.04	
**Chest sounded wheezy during or after exercise past 12 months**	1.21 (1.01, 1.46)	0.04	9656	1.16 (0.93, 1.46)	0.18	8553
**Dry cough at night past 12 months**	1.21 (1.04, 1.41)	0.02	9652	1.23 (1.04, 1.46)	0.02	8552
**In past 12 months, been told by a doctor, nurse, or other health professional that you have asthma**	0.99 (0.79, 1.25)	0.95	9700	1.13 (0.85, 1.50)	0.39	8583

Note: Unadjusted and adjusted odds ratios are from univariable and multivariable logistic models of each respiratory outcome in the first column as a function of electronic nicotine product use in past 12 months (yes/no). The adjusted association was estimated from models adjusting for age, sex, race/ethnicity, parent education, tobacco used by other household members, rules about combustible tobacco product use inside home, lifetime number of cigarettes used, and lifetime number of cigars used. “*n* in the model” differs from overall *n* due to missing data on variables in the model.

**Table 3:** Unadjusted and adjusted association between electronic nicotine product use and using asthma medications in past 12 months among youth who ever had asthma (*n* = 1917).

	Unadjusted Results			Adjusted Results		
Outcome	OR (95% CI)	*p*-Value	*n* in the Model	AOR (95% CI)	*p*-Value	*n* in the Model
Used controller/long-acting inhaler regularly past 12 months	0.98 (0.60, 1.60)	0.93	1891	1.04 (0.64, 1.68)	0.88	1703
Used quick-relief inhaler regularly past 12 months	0.94 (0.71, 1.25)	0.67	1894	1.08 (0.79, 1.48)	0.62	1704

Note: Results are from logistic models with asthma medication use (controller/long-acting inhaler, quick-relief inhaler) in past 12 months as the outcome and electronic nicotine product use (yes/no) in past 12 months as primary predictor. The adjusted association was estimated from models adjusting for age, sex, race/ethnicity, parent education, tobacco used by other household members, rules about combustible tobacco product use inside home, lifetime number of cigarettes used, and lifetime number of cigars used. “*n* in the model” differs from overall *n* due to missing data on variables in the model.

## Data Availability

National Addiction & HIV Data Archive Program (NAHDAP) PATH Wave 3 public user files. Available online: https://www.icpsr.umich.edu/web/NAHDAP/studies/36498 (accessed on 2 February 2021).
